# Supplementary material for: Early Detection and Monitoring of Gastrointestinal Infections Using Syndromic Surveillance: A Systematic Review
Source: Int J Environ Res Public Health. 2024 Apr 17;21(4):489. doi: 10.3390/ijerph21040489 (PMC11050429; doi:10.3390/ijerph21040489)
Supplement: Supplementary file 1 [file ijerph-21-00489-s001.zip › ijerph-2879961-supplementary.pdf]

Supplementary Materials

Supplementary Table S1: search strings used in the systematic review

Search string: PubMed	*Search string: Ovid/Medline*	*Search string: Scopus*	*Search string: CINAHL*
("GI activity" OR "gastrointestinal infection" OR "gastroenteritis" OR "Diarrhoea" OR "Vomiting" OR "diarrhea" OR "vomit") AND ("Syndrome surveillance" OR "Syndromic Surveillance" or "Real time surveillance" or "real-time surveillance")<br>This search string was altered to fit the syntax appropriate for each database. In Medline, this was:<br>Gastrointestinal Diseases/ or Gastrointestinal infection.mp.<br>GI activity.mp.<br>Diarrh?ea.mp.<br>Vomiting/ or vomit.mp.<br>Syndromic surveillance.mp.<br>real?time surveillance.mp.<br>real time surveillance.mp.	Gastrointestinal Diseases/ or Gastrointestinal infection.mp.<br>GI activity.mp.<br>Diarrh?ea.mp.<br>Vomiting/ or vomit.mp.<br>Syndromic surveillance.mp.<br>real?time surveillance.mp.<br>real time surveillance.mp.<br>early detection.mp.<br>1 or 2 or 3 or 4<br>5 or 6 or 7<br>10 and 11	Syndromic AND Surveillance AND Gastrointestinal<br>Syndromic AND Surveillance AND Diarrhoea<br>(real-time AND surveillance)) AND (gastrointestinal AND infection)<br>("GI activity" OR "gastrointestinal infection" OR "gastroenteritis" OR "Diarrhoea" OR "Vomiting" OR "diarrhea" OR "vomit") AND ("Syndrome surveillance" OR "Syndromic Surveillance" or "Real time surveillance" or "real-time surveillance")	Syndromic surveillance in title / all text<br>AND Gastrointestinal infection in all text<br>AND diarrhoea or diarrhea in all text<br>Real-time surveillance<br>And gastrointestinal<br>Syndromic surveillance<br>And gastroenteritis
**Search results = 161**	**Search results = 266**	**Search results = 1,286**	**Search results = 104**

Supplementary Table S2: surveillance systems included in the systematic review illustrating the primary aim/goal of the system

Author/year	Primary aim of syndromic surveillance system
Armistead 2022	To examine trends in outpatient medical care seeking behavior for acute gastroenteritis (AGE) in 2020 compared with the that of previous 3 years.
Ahn 2010	To compare the data from the emergency department syndromic surveillance system of Korea in detection and reporting of acute diarrheal syndrome (mass type) with the data from the Korea Food and Drug Administration.
Balter 2005	To review diarrhea and vomiting syndromes and to determine if any unreported outbreaks were detected.
Bounoure 2020	syndromic surveillance of medicalized acute gastroenteritis mAGE.
Brottet 2015	To identify and monitor outbreaks of influenza, gastroenteritis, and chicken pox, and to characterize circulating influenza viruses.
Caillère 2013	To investigate a major outbreak of gastroenteritis in Réunion Island in 2012 and identify the presence of G12 rotavirus on the island.
Cho 2021	To analyze the trend and characteristics of acute viral gastroenteritis.
Delespierre 2018	To assess influenza and acute gastroenteritis (AGE) syndromic data.
Donaldson 2022	To determine if cases and outbreaks in children could provide an early warning of seasonal norovirus before cases start increasing in older, more vulnerable age groups.
Edelstein 2014	To assess norovirus surveillance and response in Sweden.
Enserink 2015	To estimate the proportion of day-care attendees experiencing gastroenteritis that could be attributed to a range of enteropathogens circulating in day care in the Netherlands in 2010–2013.
Flamand 2008	To assess a large part of episodes of illness that do not require hospital admissions or the identification of an etiologic agent.
Gerstel 2009	To identify the responsible organisms for diarrhoeal illnesses.
Greene 2012	To identify potential outbreaks generated from Electronic Medical Records.
Heffernan 2004	Routinely collected chief complaint information in emergency department to detect disease outbreaks early.
Henry 2004	To assess whether nurse advice hotline data would be able to predict the syndrome diagnoses made during subsequent Kaiser Permanente of the Mid-Atlantic States syndromic outpatient office visits.
Hripcsak 2009	To identify influenza-like illness and gastrointestinal infectious disease in ambulatory electronic health record data from a network of community health centers.
Hughes 2020	National ED syndromic surveillance system to assess the continued impact of the UK national RV programme.
Kim 2023	To analyze the trend and characteristics of acute viral gastroenteritis.
Love 2023	To establish the impact of the COVID-19 outbreak response on gastrointestinal (GI) infection trends
Loveridge 2010	To investigate whether NHS Direct vomiting calls can be used as a reliable indicator of norovirus activity and, if so, whether the increase in calls precedes the epidemic of hospital outbreaks.
Lucaccioni 2021	To identify hospital episodes, deaths of rotavirus and acute gastroenteritis.
Muchaal 2015	To assess the timeliness and accuracy of pharmacy sales data for both respiratory and gastrointestinal infections and to determine its utility in supporting the surveillance of gastrointestinal illness.
Nisavanh 2022	To describe trends in acute gastroenteritis indicators.
Olson 2020	To analyze the response of rotavirus dynamics to infant vaccination.
Ondrikova 2023	To predict norovirus activity across a range of age groups across England.
Rodriguez 2007	To characterize emergency department (ED) visits for gastroenteritis by season and age and develop a predictive model.
Smith 2007	An established primary care-derived database, that provide timely and local information on trends in community illness and prescribing.
Tanabe 2018	The benefits of the (Nursery) School Absenteeism Surveillance System, (N)SASSy, as an infection control measure by a public health center.

Supplementary Table S3: quality assessment of studies using the Joanna Briggs Institute (JBI) critical appraisal checklist for qualitative research

**Study**	**Is there agreement between the stated philosophical perspective and the research methodology?**	**Is there agreement between the research methodology and the research question or objectives?**	**Is there agreement between the research methodology and the methods used to collect data?**	**Is there agreement between the research methodology and the representation and analysis of data?**	**Is there agreement between the research methodology and the interpretation of results?**	**Is there a statement locating the researcher culturally or theoretically?**	**Is the influence of the researcher on the research, and vice-versa, addressed?**	**Are participants, and their voices, adequately represented?**	**Is the research ethical according to current criteria or, for recent studies, and is there evidence of ethical approval by an appropriate body?**	**Do the conclusions drawn in the research report flow from the analysis, or interpretation, of the data?**	**Overall appraisal**
Armistead 2022	✓	✓	✓	✓	✓	X	X	X	✓	✓	High
Ahn 2010	✓	✓	✓	✓	✓	X	X	✓	X	✓	High
Balter 2005	✓	✓	✓	✓	✓	X	X	X	X	✓	Medium
Bounoure 2020	✓	✓	X	X	✓	X	X	X	X	✓	Low
Brottet 2015	✓	X	✓	X	✓	X	X	X	X	✓	Low
Caillère 2013	✓	X	✓	✓	✓	X	X	X	X	✓	Medium
Cho 2021	✓	✓	✓	✓	✓	X	X	✓	✓	✓	High

Delespierre 2018	✓	✓	✓	✓	✓	X	X	✓	✓	✓	High
Donaldson 2022	✓	✓	✓	✓	✓	X	X	✓	✓	✓	High
Edelstein 2014	✓	✓	✓	✓	✓	X	X	✓	X	✓	High
Enserink 2015	✓	✓	✓	X	✓	X	X	✓	✓	✓	High
Flamand 2008	✓	✓	✓	✓	✓	X	X	✓	X	✓	High
Gerstel 2009	✓	✓	✓	✓	✓	X	X	X	X	✓	Medium
Greene 2012	✓	✓	✓	✓	✓	X	X	X	X	✓	Medium
Heffernan 2004	✓	X	✓	✓	✓	X	X	X	X	✓	Medium
Henry 2004	✓	✓	✓	✓	✓	X	X	X	X	✓	Medium
Hripcsak 2009	✓	X	X	X	X	X	X	X	X	X	Low
Hughes 2010	✓	✓	✓	✓	✓	X	X	✓	✓	✓	High
Kim 2023	✓	✓	✓	✓	✓	X	X	✓	✓	✓	High
Love 2022	✓	✓	✓	✓	✓	X	X	✓	✓	✓	High
Loveridge 2010	✓	✓	✓	✓	✓	X	X	✓	X	✓	High
Lucaccioni 2021	✓	✓	✓	✓	✓	X	X	X	X	✓	Medium
Muchaal 2015	✓	✓	✓	✓	✓	X	X	X	X	✓	Medium

Nisavanh 2022	✓	✓	✓	✓	✓	X	X	X	✓	✓	High
Olson 2020	✓	X	X	✓	✓	X	X	X	X	✓	Low
Ondrikova 2023	✓	✓	✓	✓	✓	X	X	✓	✓	✓	High
Rodriguez 2007	✓	✓	✓	✓	✓	X	X	X	X	✓	Medium
Smith 2007	✓	✓	✓	✓	✓	X	X	X	X	✓	Medium
Tanabe 2018	✓	✓	✓	✓	✓	X	X	X	✓	✓	High
